# Selected micronutrient status of school-aged children at risk of *Schistosoma haematobium* infection in suburban communities of Nigeria

**DOI:** 10.4102/ajlm.v12i1.2034

**Published:** 2023-05-31

**Authors:** Samson E. Olerimi, Ehitare I. Ekhoye, Oriasotie S. Enaiho, Alexander Olerimi

**Affiliations:** 1 Department of Medical Biochemistry, Faculty of Basic Medical Sciences, Ambrose Alli University, Ekpoma, Nigeria; 2 School of Biomedical Sciences, Nottingham Trent University, Nottingham, United Kingdom; 3 Department of Physiology, Faculty of Basic Medical Sciences, Edo State University, Uzairue, Nigeria; 4 Department of Mathematics, Faculty of Natural Sciences, Ambrose Alli University, Ekpoma, Nigeria

**Keywords:** micronutrients, *Schistosoma haematobium*, schistosomiasis, school-age children, zinc

## Abstract

**Background:**

The parasite *Schistosoma haematobium* causes urogenital schistosomiasis, a chronic infectious disease that occurs mainly among school-age children.

**Objective:**

The prevalence of *S. haematobium* infection and level of intensity relative to age, gender and status of selected serum micronutrients among school-age children were investigated in suburban communities in Bekwarra, Nigeria.

**Methods:**

This cross-sectional school-based study randomly recruited 353 children aged between 4 and 16 years from five elementary schools between June 2019 and December 2019. We gathered socio-demographic data about each child using a semi-structured questionnaire. Blood samples were collected for micronutrient analysis and urine samples were collected for assessment of *S. haematobium* infection.

**Results:**

A total of 57 (16.15%) school-age children were infected with *S. haematobium*. Girls (*n* = 34; 9.63%) were more frequently infected than boys (*n* = 23; 6.52%). Infection was most frequent among children aged 8–11 years (*n* = 32; 23.19%) and was significantly associated with age (*p* = 0.022) and gender (*p* < 0.001). Serum levels of iron, calcium, copper and zinc among infected children were significantly lower than those of non-infected children. Intensity of infection was negatively associated with iron (*r* = −0.21), calcium (*r* = −0.24), copper (*r* = −0.61; *p* < 0.001) and zinc (*r* = −0.41; *p* < 0.002).

**Conclusion:**

This study showed that *S. haematobium* infection adversely impacted the micronutrient status of school-age children in suburban Nigeria. Measures to lower the prevalence of schistosomiasis among school-age children, including efficient drug distribution, education campaigns and community engagement, are necessary.

**What this study adds:**

This research emphasises the significance of implementing infection prevention and control interventions to mitigate the transmission and prevalence of schistosomiasis among school age children.

## Introduction

Micronutrients are essential in health and development. The spread of helminth infestations and micronutrient deficiencies overlaps in tropical and subtropical regions.^1,2^ Schistosomiasis, also known as bilharzia, is a neglected tropical disease caused by trematodes classified in the genus *Schistosoma*.^3^ It manifests as intestinal and/or urinary disease in the gastrointestinal or genitourinary tract.^4^ Global estimates in 2019 suggest that schistosomes infect 240 million people, while about 700 million persons are at risk annually.^5^

Most countries have successfully reduced schistosomiasis spread and infection rates; however, a large burden of infection persists in Africa, especially in tropical regions.^6,7^ Nigeria has one of the highest prevalences of schistosomiasis in sub-Saharan Africa,^8,9^ with over 101 million people at risk and an estimated 29 million people infected, of which 16 million are children.^10^ *Schistosoma haematobium*, the most extensively distributed *Schistosoma* species in Nigeria, is found mainly in the country’s southern regions.^11^ Schistosomiasis is associated with poverty, lack of or insufficient clean water, poor sanitation, and hygiene.^12^ It has been extensively documented in Nigeria that schistosomiasis mortality and morbidity are highest among children of school-age.^13,14^

Schistosomiasis is most prevalent among school-age children.^15^ It is transmitted by the *Bulinus* snail, which is the intermediate host for *S. haematobium*.^16^ The disease spread among school-age children aged 10–15 years may primarily be attributable to frequent contact with organism-contaminated water in endemic areas.^17^ Parasite infection negatively impacts the nutritional status of children by inducing loss of appetite and increasing nutrient waste owing to blood loss, vomiting, and diarrhoea.^18^

Micronutrients, such as copper, zinc, iron, and calcium, are essential for the maintenance of life. Micronutrient deficiencies of iron, copper, manganese and zinc exacerbate *Schistosoma* spp. infections and other parasitic ailments associated with haemorrhage.^19^ These micronutrients have numerous roles in the complex enzyme systems that perform various biological processes. The trace element zinc has catalytic, structural and regulatory functions,^20^ and its deficiency is responsible for 20% of all child deaths worldwide.^21^ Symptoms of zinc deficiency include chronic diarrhoea, coeliac disease, inflammatory bowel disease, ileostomy, alcoholic cirrhosis, and haemolytic anaemia.^22^ Copper protects cells from free-radical damage by acting as an antioxidant adjuvant.^23^ Copper deficiency is uncommon; however, it has been reported in preterm neonates, cowmilk-fed infants, and infants recovering from diarrhoea-caused malnutrition.^24^ Copper deficiency results in anaemia, neutropenia, growth retardation, altered glucose and lipid metabolism, and higher infection rates.^25^ Iron is involved in the formation of haemoglobin, myoglobin and catalase.^26^ Its deficiency is believed to affect around a quarter of the world’s population. Infants aged 4–24 months, school-age children, women and girls, adolescents, and pregnant and breastfeeding mothers are the most affected.^27^ Calcium is essential for many biological activities, such as bone and tooth structure, signal transmission, muscle contraction, enzyme regulation, and blood coagulation.^28^ There is evidence that appropriate calcium intake is vital during childhood and adolescence, and that adequate calcium during these life stages is crucial.^29^ Calcium depletion is associated with rickets and osteomalacia, pre-eclampsia, osteoporosis, and preterm delivery, particularly in developing countries.^30^

Schistosomiasis is a serious disease that mostly affects children, especially in impoverished communities with limited health care and a lack of access to safe drinking water. Malnutrition promotes susceptibility to infection due to lower appetite and increased nutritional requirements, resulting in a vicious cycle that can have adverse effects on the nutrition status of children and adolescents.

The effects of *S. haematobium* on school-age children have been established in sub-Saharan Africa and some regions of Nigeria. Data on the impact of *Schistosoma* spp. on the micronutrient levels of school children in Bekwarra are sparse. The current estimate of the population of Bekwarra Local Government Area (LGA) is 134 108 as of 2017, using the 2017 annual growth rate of 2.4% in the Cross River State. The Bekwarra LGA is a typical rural area with a tropical climate, characterised by a hot, dry season (December–February) and a cooler, wet season (April–November). Most communities in Bekwarra LGA are rural settlements that are encircled by a multitude of freshwater ecosystems, streams and rivers. Residents depend mostly on rainfall, wells, rivers and streams for their water needs. Thus, there is the possibility that in these riverine settlements the *S. haematobium* vector, the *Bulinus* snail, is endemic. Moreso, people’s reliance on streams and rivers for consumption and household water may increase their exposure to *S. haematobium* infection.

This study is thus aimed to investigate the serum levels of selected micronutrients in school-age children with urinary schistosomiasis in Bekwarra LGA, Cross River State, Nigeria.

## Methods

### Ethical considerations

Ethical clearance to conduct this study was obtained from the Cross River Ministry of Health Research Ethics Committee (No. CRSMOH/RP/REC/2017/806). The teachers helped to interview the participants. They also assisted the research staff in communicating the research objectives to participants. During the daily weekday child pick-up, each legal guardian and/or parent whose children verbally assented to participate was contacted separately. Guardians and/or parents were informed verbally, either in English or the local dialect of the research’s objectives and protocols, and subsequently administered an informed consent form that included items that were discussed with them. The consent forms given were either returned immediately or at the next day’s drop-off. Only school-age children with verbal and written agreement from their legal guardian and/or parent were included in the study. Each participant’s information was handled in strict confidentiality. All the questionnaires and results were coded (participants did not write their names on the questionnaires and all responses were typed into a password-locked software program) to meet the principle of confidentiality. Parents and/or guardians whose children tested positive for *S. haematobium* were notified and advised to take their children to the Primary Health Centres, Bekwarra, for treatment. A follow-up by the research team through the Headmaster or head-mistress and class teachers of the affected school ensured that all infected school-age children were treated successfully.

### Study design and areas

Adopting a systematic random-sampling approach, this cross-sectional study was carried out in five separate primary schools in the communities of Nyanya, Abouchichie, Otupuru, and Ukpah in Bekwarra LGA, Cross River State, Nigeria for 6 months, from June 2019 to December 2019.

### Study sampling

The sample size was determined using the single population proportion formula. Based on earlier data on schistosomiasis in communities within Bekwarra LGA,^31^ the sample size was computed with a prevalence of 29.5%. The sample size was 325 with a precision of 0.05 (5%). The statistical power utilised was 95%. Therefore, children within the age range of 4–16 years attending primary schools within Bekwarra LGA, Cross River State, who in the preceding 6 months had not received any anti-helminth treatment, were selected from the communities under focus. A response rate of 98.06% was achieved with the participation of 353 school-age children out of the original target population of 360. All the participants had lived in their respective communities for the past 2 years. All participants who completed the questionnaire and delivered their samples of urine for examination were selected for this study. Menstruating girls were exempted.

### Collection of samples and data

#### Questionnaire administration

A structured questionnaire was administered to each child by pre-trained research assistants who were well supervised. The questionnaire contained sections that requested information on aspects of socio-demography.

#### Parasitology survey

A 10 mL urine sample was collected from each child between 10:00 and 14:00 in a designated private area of the school to assess the presence of a high *S. haematobium* egg load, using the sedimentation quantitative technique.^32^ Then, 0.2 mL of 37% formalin was added to preserve the urine sample, which was then transported to a dedicated facility (a few metres from the site of collection) within the school in a cooler with ice packs, for examination to determine the presence and parasitic load of *S. haematobium*. The urine samples were centrifuged at 5000 revolutions per minute for 5 min. The supernatant obtained after the centrifugation was then discarded, leaving only the sediment. The sediment was then placed on a cleaned glass slide, then covered with a coverslip. These slides were microscopically observed using a × 40 objective lens for *S. haematobium* eggs, characterised by a terminal spine. The presence of *S. haematobium* eggs indicates a positive sample and was expressed as the number of eggs per 10 mL of the sample. The parasitic load of *S. haematobium* was categorised as mild (< 50 eggs/10 mL of urine) or heavy (≥ 50 eggs/10 mL of urine).^33^ A few drops of saponin solution were applied to samples with visible haematuria to enhance clarity for microscopy.^34^

#### Serum micronutrient assay

Participants’ blood specimens were collected to determine micronutrient levels. Trace element-free syringes, stainless steel needles, and special trace element tubes made of polypropylene (Becton Dickinson, Franklin Lakes, New Jersey, United States) were used. All tubes used were stored in cool, dark boxes (0 °C – 4 °C). The separation of sera from blood cells was done by centrifugation at 4000 revolutions per minute for 10 min at 4 °C, within 4 h of sample collection. Aliquots of sera obtained were stored at –70 °C until analysis. Serum samples were diluted with sterile distilled water at a 1:6 ratio. An atomic absorption spectrophotometer (Varian AA 100, Victoria, Australia) was used to measure the activity of serum zinc (at 213.9 nm), copper (324.8 nm), iron (510 nm) and calcium (422.7 nm),^35,36^ following previously published procedures.^37^ The results were then expressed in mg/dL for calcium, and μg/dL for copper, iron and zinc.

### Statistical analysis

Data analysis was done using the Statistical Package for Social Sciences software version 23.0 (SPSS, Inc., Chicago, Illinois, United States). A one-sample Kolmogorov-Smirnov test was adopted to measure data normality distribution. All micronutrient values in the serum were of normal distribution. The means for participants’ serum micronutrients levels were compared using one-way analysis of variance, followed by the Least Significant Difference post hoc test for comparison between groups. Chi-square and Fisher’s Exact tests were used to determine the relationship between infection patterns and age and gender. The correlation of two continuous variables was determined using Pearson’s test. Statistical significance was accepted as *p-*values less than 0.005 and 0.001.

## Results

This study enrolled 353 school-age children within the age range of 4–16 years (Table 1). They consist of 223 (63.17%) boys and 130 (36.83%) girls. The overall prevalence was 16.15%. A total of 43 (12.18%) children had a mild infection, while 14 (3.97%) had a heavy infection; of this group with heavy infection, 2 (1.43%) had visible urinary haematuria. *S. haematobium* prevalence was highest in children 8–10 years old (*n* = 32, 23.19%), and lowest in children 14–16 years old (*n* = 4, 7.28%). There was a significant association between the infection rate and the age of the children (*p* = 0.022). In addition, the highest rate of *S. haematobium* infection was observed in girls (*n* = 34, 26.15%), while boys had an infection rate of 23 (10.31%). A significant association was observed between the gender and infection rate (*p* < 0.001).

**TABLE 1:** Demographics and *Schistosoma haematobium* infection among school-age children from Bekwarra communities, Nigeria, June 2019 – December 2019.

Characteristics	Number examined		Number infected		*S. haematobium infection*						Visible haematuria	
					Negative		Mild		Heavy			
	*N*	%	*n*	%	*n*	%	*n*	%	*n*	%	*n*	%
**Age group (years)** †												
4–7	78	22.10	9	11.54	69	88.46	6	7.69	3	3.85	1	1.28
8–10	138	39.09	32	23.19	106	76.81	25	18.12	7	5.07	3	2.17
11–13	82	23.23	12	14.63	70	85.37	9	10.98	3	3.65	1	1.22
14–16	55	15.58	4	7.28	51	92.72	3	5.46	1	1.82	0	0.00
Total	353	100.00	57	16.15	296	83.85	43	12.18	14	3.97	5	1.43
**Gender** ‡												
Male	223	63.17	23	10.31	200	89.69	19	8.52	4	1.79	2	0.90
Female	130	36.83	34	26.15	96	73.85	24	18.46	10	7.69	3	2.31

*N*, number examined; *n*, number infected; %, percentage; χ^2^, chi-square; FET, Fisher’s exact test.

† , χ^2^ (3, *N* = 353) = 9.61, *p* = 0.022;

‡ , *p* = 0.000113, FET.

The mean calcium serum level was significantly (*p* = 0.001) lower in heavily-infected children when compared to children without infection. Serum concentrations of iron (*p* < 0.001), copper and zinc were significantly lower in children with mild *S. haematobium* infection compared with children without infection. Furthermore, there were significant reductions in serum concentrations of iron (*p* < 0.001), zinc (*p* < 0.001) and copper (*p* < 0.002) in children with heavy *S. haematobium* infection when compared to children without infection. Bivariate correlation analysis showed a significant inverse correlation between the number of *S. haematobium* eggs/10 mL in urine and the levels of serum copper (*r* = −0.61, *p* < 0.001) and zinc (*r* = −0.41, *p* < 0.002). Also, an inverse correlation was found between the number of *S. haematobium* eggs/10 mL in urine and serum calcium (*r* = −0.24) and iron (*r* = −0.21) levels, but the correlations were not statistically significant (Table 2).

**TABLE 2:** Serum levels of selected micronutrients in school-age children with *S. haematobium* infection from Bekwarra communities, Nigeria, June 2019 – December 2019.

Micronutrients	*S. haematobium* infection status					Correlation	*p*
	Negative	Mild (< 50 eggs/10 mL)		Heavy (≥ 50 eggs/10 mL)			
		Mean ± s.d.	*p*	Mean ± s.d.	*p*		
Calcium (mg/dL)	8.72 ± 3.79	7.56 ± 4.48	0.014	6.00 ± 4.30	0.001	−0.24	0.086
Iron (μg/dL)	97.63 ± 42.90	66.21 ± 33.06	< 0.001	37.98 ± 20.48	< 0.001	−0.21	0.012
Copper (μg/dL)	121.59 ± 32.49	91.99 ± 34.86	< 0.001	70.29 ± 21.97	0.002	−0.61	< 0.001
Zinc (μg/dL)	88.27 ± 27.42	53.05 ± 27.01	< 0.001	36.47 ± 17.53	< 0.001	−0.41	0.002

## Discussion

This study provides baseline epidemiological data on urogenital schistosomiasis in school-age children in the Bekwarra villages of Cross River, Nigeria. Our findings suggest that urogenital schistosomiasis is a prevailing parasitic infection in our study setting, similar to reports of it being a significant cause of morbidity in Nigerian children in some riverine communities.^38,39^ Overall, the prevalence in our study area is 16.15%, which is almost double the national Nigerian average of 8.1% recorded in 2012.^11^ Our results are consistent with previous findings from numerous endemic regions in Nigeria ^40,41^ and other endemic countries such as Tanzania, Angola, Malawi, and Burkina Faso.^42,43,44,45^ However, the severity and incidence of infection in our study are lower compared to data from several regions of Nigeria and other sub-Saharan countries.^46,47,48^ These studies had a larger participant size, consequently increasing the proportion of exposed and infected participants.

The age group with the highest burden of infection in this study is those children younger than 11 years old, and the infection prevalence peaked at 8–11 years, similar to reports by Olalubi and Olukunle.^49^ Our observation is most likely because children within this age range are frequently involved in water-contact activities (e.g., swimming, animal watering, and washing), and perhaps, this age group may not have been included in the recent campaign and distribution of preventive chemotherapy conducted in Cross River State.^50^ The study showed that boys were frequently exposed to *S. haematobium* risk factors; nonetheless, infection prevalence was greater in girls. The higher prevalence in girls might be because the girls may routinely engage in house chores and associated activities, such as dish and cloth washing, fetching water from the stream for cleaning, bathing, and other domestic purposes, as well as the inherent anatomical differences between girls and boys.^51^ The greater prevalence rate found in girls compared to boys is comparable with previous findings for *S. haematobium* infection in Uganda^52^ and Nigeria,^53^ where female subjects had a higher infectious burden than males. However, the findings of this study contrast those of other comparable studies conducted in Mauritania,^54^ Senegal,^55^ Benin and Ethiopia.^56^ The distinction may be explained by differences in sociocultural elements, where boys are more likely to engage in water-contact activities including swimming and bathing, fishing, farming, and watering cattle.

Parasitic infections can cause nutrient loss, leading to poor nutritional status, which increases susceptibility to infection, creating a vicious cycle.^57^ We observed a negative correlation between the intensity of *S. haematobium* infection and micronutrient status. This observation indicates that the concentrations of calcium, iron, copper and zinc diminish as the infection intensity increases in school children. Although the report of Osazuwa et al.^58^ confirmed our observed negative correlation between iron and *S. haematobium* infection, previous studies found no correlation between micronutrient status for calcium, copper and zinc with helminth infection.^59,60,61,62^ Thus, the explicit impact of parasitic infection on nutrient loss remains unclear.

### Limitations

Confounders, such as other *Schistosoma* species and microorganisms, were not examined; however, they might have influenced the data distribution in the research. The inability to analyse the snail host’s water sources and objectively quantify school-age children’s water-contact activities may restrict the interpretation of findings. Another limitation of the study was the difficulty in obtaining guardian permission due to superstitious concerns regarding the use of participants’ urine samples.

### Conclusion

This study found a relatively low incidence of urinary schistosomiasis infection. Providing potable water in the study communities will reduce exposure to infected water, thereby reducing transmission. There were significant differences established between sex and age and urinary schistosomiasis infectious diseases. These findings showed that urogenital schistosomiasis causes considerable morbidity and may be one of the contributing reasons to malnutrition in school-age children residing in Bekwarra LGA, Cross River State. Routine mass treatment will decrease morbidity and gradually eradicate the disease in the communities. Therefore, there is a need for continual disease evaluation, provision of basic health education, implementation of broad-based health care policy, and development of socioeconomic infrastructures including providing safe and clean water for drinking through government agencies and cooperating organisation collaborations. These are essential in endemic areas to reduce infection transmission.
